# Impact of *UGT2B7 ^His268Tyr ^*polymorphism on the outcome of adjuvant epirubicin treatment in breast cancer

**DOI:** 10.1186/bcr2894

**Published:** 2011-06-09

**Authors:** Sumit Parmar, Julia Carolin Stingl, Ariana Huber-Wechselberger, Alexander Kainz, Wilfried Renner, Uwe Langsenlehner, Peter Krippl, Jürgen Brockmöller, Elisabeth Haschke-Becher

**Affiliations:** 1 Institute of Pharmacology of Natural Products and Clinical Pharmacology, University Ulm, Helmholtzstrasse 20, Ulm, 89081, Germany; 2 Institute of Medical and Laboratory Diagnostics, Elisabethinen Hospital Linz, Fadingerstrasse 1, Linz, 4020, Austria; 3 Department of Nephrology and Dialysis, Medical University Vienna, Währingergürtel 18-20, Vienna, 1090, Austria; 4 Clinical Institute of Medical and Laboratory Diagnostics, Medical University Graz, Auenbruggerplatz 15, Graz, 8036, Austria; 5 Department of Internal Medicine, Hospital of Fürstenfeld, Krankenhausgasse 1, Fürstenfeld, 8280, Austria; 6 Department of Clinical Pharmacology, University Göttingen, Robert-Koch-Strasse 40, Göttingen, 37075, Germany; 7 Christian Doppler Clinic, Private Paracelsus Medical University Salzburg, Ignaz Harrerstrasse 79, Salzburg, 5020, Austria

## Abstract

**Introduction:**

Epirubicin is a common adjuvant treatment for breast cancer. It is mainly eliminated after glucuronidation through uridine diphosphate-glucuronosyltransferase 2B7 (UGT2B7). The present study aimed to describe the impact of the *UGT2B7^His268Tyr ^*polymorphism on invasive disease-free survival in breast cancer patients after epirubicin treatment.

**Methods:**

This is a pharmacogenetic study based on samples collected from 745 breast cancer patients of the Austrian *T*umor of breast tissue: *I*ncidence, *G*enetics, and *E*nvironmental *R*isk factors (*TIGER*) cohort who did not present metastases at baseline. This cohort included 205 women with epirubicin-based combination chemotherapy, 113 patients having received chemotherapy without epirubicin and 427 patients having received no chemotherapy at all. Of the epirubicin-treated subgroup, 120 were subsequently treated with tamoxifen. For all women *UGT2B7^His268Tyr ^*was genotyped. Invasive disease-free survival was assessed using Kaplan-Meier and Cox's proportional hazard regression analysis.

**Results:**

Among the 205 epirubicin-treated patients, carriers of two *UGT2B7^268Tyr ^*alleles had a mean invasive disease-free survival of 8.6 (95% confidence interval (CI) 7.9 to 9.3) years as compared to 7.5 (95% CI 6.9 to 8.0) years in carriers of at least one *UGT2B7^268His ^*allele (adjusted hazard ratio (HR) = 2.64 (95% CI 1.22 to 5.71); *P *= 0.014). In addition, the impact of the *UGT2B7^His268Tyr ^*polymorphism became even more pronounced in patients subsequently treated with tamoxifen (adjusted HR = 5.22 (95% CI 1.67 to 26.04); *P *= 0.015) whereas no such difference in invasive disease-free survival was observed in patients not receiving epirubicin.

**Conclusions:**

Breast cancer patients carrying the *UGT2B7^268Tyr/Tyr ^*genotype may benefit most from adjuvant epirubicin-based chemotherapy. These results warrant confirmation in further studies.

## Introduction

Anthracyclines are commonly used for neoadjuvant or adjuvant chemotherapy of locally advanced breast cancer [1,2]. They act by inhibition of topoisomerase II alpha and generation of reactive oxygen species eventually resulting in cell cycle arrest and apoptosis [3,4]. Epirubicin is a semisynthetic derivative of doxorubicin preferentially used in breast cancer treatment. It is extensively metabolized in the liver to epirubicinol by aldo-keto reductase and in addition aglycones of epirubicin and epirubicinol are formed [5]. The major inactivation pathway for epirubicin and epirubicinol is glucuronidation catalysed by UDP-glucuronosyltransferases (UGT). Studies using human liver microsomes, expressing UGT1A1, UGT1A3, UGT1A4, UGT1A6, UGT1A9, UGT2B7 and UGT2B15 revealed that UGT2B7 uniquely converts epirubicin to its glucuronide [6]. In addition, also the active metabolites of tamoxifen are eliminated by UGT2B7 mediated glucuronidation [7].

The *UGT2B7 *gene is polymorphic with a frequent non-synonymous variant 802 C > T leading to a histidine to tyrosine substitution in codon 268 (His268Tyr). The functional impact of this polymorphism is unclear as studies have shown lower [8-12], similar [13-15], and even higher enzyme activity of the UGT2B7^268Tyr ^isoform [16-18]. Two *in vitro *studies showed no impact of the *UGT2B7^His268Tyr ^*genotype on the epirubicin glucuronide formation [6,19]. However, the presently available *in vitro *studies cannot finally clarify, if variants linked with the *UGT2B7^His268Tyr ^*polymorphism may modulate up- or downregulation of the enzyme under the conditions of adjuvant or neoadjuvant chemotherapy and *in vitro *models can, of course, never fully resemble the *in vivo *conditions [19-22]. *UGT2B7 *haplotype analysis revealed six promoter variants (-1306 G > A, -1299C > T, -1112C > T, -900A > G, -327G > A and -161C > T) which modulate UGT2B7 promoter activity and are in perfect linkage disequilibrium with the *UGT2B7^His268Tyr ^*variant [19,20,22,23]. In contrast, for 4-hydroxy-tamoxifen and endoxifen, the active metabolites of tamoxifen, a decreased glucuronidation activity was shown for the UGT2B7^268Tyr ^isoform *in vitro *[9].

Here we present a pharmacogenetic (PGt) analysis of the Austrian *T*umor of breast tissue: *I*ncidence, *G*enetics, and *E*nvironmental *R*isk factors (*TIGER*) study [24,25] to explore the effect of the *UGT2B7^His268Tyr ^*polymorphism on invasive disease-free survival after epirubicin treatment.

## Materials and methods

### Study design

The TIGER study was a cohort study to investigate genetic and environmental risk factors relevant to the onset and course of breast cancer [24,25]. As described earlier [26], the complete cohort consisted of 804 consecutive women with histologically confirmed breast cancer and no other cancer diagnosis, treated at the Division of Oncology, Department of Internal Medicine, Medical University Graz, Austria between January 2000 and September 2004. For the purpose of this PGt study, patients presenting with metastases already at baseline (stage IV) or not having biomaterial available for PGt analyses were excluded. At stage IV host genetic polymorphisms were considered much less relevant for survival and sample size in this subgroup was too small (*n *= 38) to allow adjustment or stratified analysis for this stage of disease. Patients were regularly followed-up with clinical examinations, laboratory (including CEA and CA15-3), radiological (bone scan, liver scan, chest X-ray, and mammograms), and gynecological analyses at three-year intervals until 2010 [24].

The study complied with the Declaration of Helsinki and was performed according to the Austrian Gene Technology Act. The protocol has been approved by the Ethical Committee of the Medical University Graz. Written informed consent was obtained from all participating subjects.

### UGT2B7 genotyping

DNA was isolated from venous blood (Qiamp DNA Mini-Kit, Qiagen, Hilden, Germany). Genotyping for *UGT2B7 802 C > T *was performed blinded to the clinical outcomes using the validated TaqMan^® ^SNP Genotyping Assay (C__32449742_20, Applied Biosystems, Foster City, CA, USA). Real-time PCR reactions were set up in a final volume of 12.5 μl containing TaqMan^® ^Genotyping Master Mix, 20 × SNP Genotyping Assay (Applied Biosystems, Foster City, CA, USA) distilled water and 3 to 20 ng genomic DNA. PCR amplification was carried out using the Real Time PCR System 7300 (Applied Biosystems) under the following conditions: 10 minutes 95°C enzyme activation followed by 40 cycles at 92°C for 15 s and at 60°C for 1 minute.

### Statistical analysis

Invasive disease-free survival was defined as the time from diagnosis to any local, regional, or distant recurrence, metastases or contralateral breast cancer or death from any cause, but excluded second primary invasive cancer to consider only events which may reflect efficacy of initial epirubicin drug treatment [27]. Kaplan-Meier estimates were calculated and the log-rank test was used to compare invasive disease-free survival of different *UGT2B7^His268Tyr ^*variant carriers after (1) adjuvant chemotherapy with epirubicin, (2) adjuvant chemotherapy with epirubicin and tamoxifen, (3) adjuvant chemotherapy other than epirubicin, or (4) no adjuvant chemotherapy at all. In the latter two subgroups no difference was expected and thus served as controls. Cox's proportional hazard modeling was used for subsequent multivariate analyses with adjustments for clinical prognostic factors such as tumor size, age at diagnosis, nodal status, and histological grade. Proportional hazards were assessed by Schoenfeld residuals as well as by including the interaction terms of covariates with time into the model. Possible differences in patient characteristics between the respective treatment and genotype groups were assessed using Chi-square test, Kruskal-Wallis test or Mann-Whitney test. All tests were two-sided, performed at a significance level of 0.05 using SPSS 17.0 (SPSS Inc., Sunnyvale, CA, USA).

## Results

### Study population

**Figure 1:**
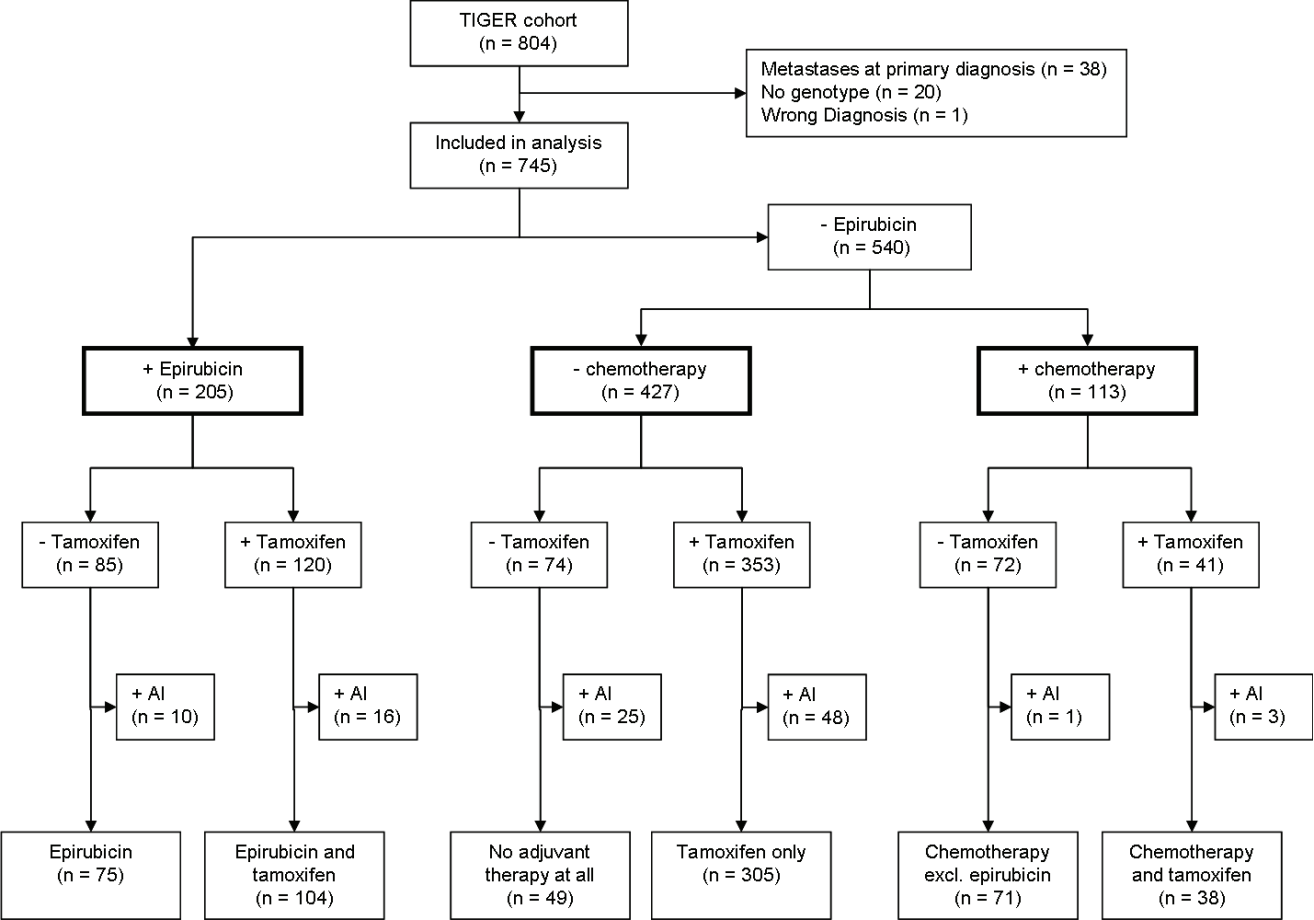
**Patient flow chart based on the original TIGER cohort**. AI, aromatase inhibitor, TIGER, Austrian tumor of breast tissue: incidence, genetics, and environmental risk factors study.

Of the original 804 patients of the TIGER cohort, 59 patients were excluded from this analysis due to metastases already at baseline, the lack of material for PGt analyses, or the revision of the breast cancer diagnosis during follow-up (Figure 1). Of the remaining 745 patients, 205 received epirubicin-based combination chemotherapy, 113 combination chemotherapy without epirubicin (mostly cyclophosphamide, methotrexate and fluorouracil), and 427 no adjuvant chemotherapy at all. Of the 205 epirubicin-treated patients, 120 subsequently also received tamoxifen (Figure 1).

### Invasive disease-free survival by treatment

Patients who underwent adjuvant chemotherapy significantly differed from the remaining patients in clinical prognostic factors such as age, tumor size, grade, and nodal status (Table 1). Mean invasive disease-free survival times differed accordingly, with 7.9 years (95% confidence interval (CI) 7.4 to 8.3) in epirubicin-treated patients, 6.9 years (95% CI 6.1 to 7.6) in patients with adjuvant therapy other than epirubicin, and 9.1 years (95% CI 8.8 to 9.4) in the remaining patients. Corresponding rates of patients with disease progression during a mean follow-up period of 6.6 ± 2.0 years were 27.8%, 46.9%, and 18.3%, respectively.

**Table 1:** Baseline characteristics by treatment

Adjuvant chemotherapy	Epirubicin	No epirubicin	None	P*
Total patients No. (%)	205 (27.5%)	113 (15.2%)	427 (57.3%)	
Age in years (mean, SD)	53.0 (10.5)	55.6 (12.6)	60.5 (12.0)	< 0.001
Tumor size				< 0.001
≤ T1 (≤ 20 mm)	75 (36.6%)	59 (52.2%)	296 (69.3%)	
≥ T2	127 (62.0%)	50 (44.2%)	121 (28.3%)	
unknown	3 (1.5%)	4 (3.5%)	10 (2.3%)	
Lymph nodes				< 0.001
0	32 (15.6%)	45 (39.8%)	262 (61.4%)	
≥ 1	162 (79.0%)	57 (50.4%)	131 (30.7%)	
unknown	11 (5.4%)	11 (9.7%)	34 (8.0%)	
Grade				< 0.001
1	3 (1.5%)	4 (3.5%)	45 (10.5%)	
2	63 (30.7%)	31 (27.4%)	260 (60.9%)	
> 2	128 (62.4%)	77 (68.1%)	107 (25.1%)	
Unknown	11 (5.4%)	1 (0.9%)	15 (3.5%)	

* *P*-values are indicated for the comparison of patients treated with adjuvant chemotherapy (with or without epirubicin) vs. patients not treated with any adjuvant chemotherapy.

SD, standard deviation

### Invasive disease-free survival by *UGT2B7 ^His268Tyr ^*genotype

In the entire cohort, 147 (19.7%) patients carried two histidine alleles, 352 (47.2%) were heterozygous and 246 (33.0%) carried two tyrosine-alleles. *UGT2B7^268Tyr ^*allele frequency was 57% and genotype frequencies matched Hardy Weinberg equilibrium (Chi square test, *P *= 0.27). Neither did frequencies of genotypes vary significantly among the three different treatment groups (Chi-square, *P *= 0.93) nor did epirubicin-treated carriers of different variants differ in tumor size, grade, and nodal status at baseline (Table 2). However, carriers of at least one *UGT2B7^268His ^*allele were slightly older than non-carriers at baseline (54.4 ± 10.2 versus 50.1 ± 10.7; Mann-Whitney test, *P *= 0.01).

**Table 2:** Characteristics of epirubicin-treated patients by *UGT2B7^His268Tyr^*genotype

UGT2B7^268 ^Genotype	Tyr/Tyr	His/Tyr + His/His	*P*
Number (%)	66 (32.2%)	139 (67.8%)	
Age in years (mean, SD)	50.1 (10.7)	54.4 (10.2)	*0.01*
Tumor size			*0.12*
≤ T1 (≤ 20 mm)	29 (43.9%)	46 (33.1%)	
≥ T2	35 (53.0%)	92 (66.2%)	
unknown	2 (3.0%)	1 (0.7%)	
Lymph nodes			*0.73*
0	12 (18.2%)	20 (14.4%)	
≥ 1	50 (75.8%)	112 (80.6%)	
unknown	4 (6.1%)	7 (5.0%)	
Grade			*0.62*
1	1 (1.5%)	2 (1.4%)	
2	17 (25.8%)	46 (33.1%)	
> 2	43 (65.2%)	85 (61.2%)	
Unknown	5 (7.6%)	6 (4.3%)	

SD, standard deviation

**Figure 2:**
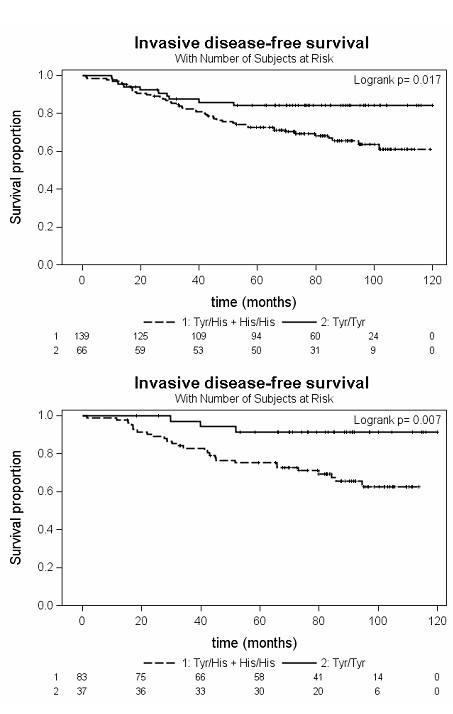
**Kaplan-Meier plots of invasive disease-free survival depending on the *UGT2B7^His268Tyr ^*genotype in epirubicin-treated patients**. (b) epirubicin-treated patients subsequently treated with tamoxifen.

In epirubicin-treated patients an association between the *UGT2B7^His268Tyr ^*polymorphism and invasive disease-free survival was observed (log-rank *P *= 0.05). By comparing carriers and non-carriers of the *UGT2B7^268His ^*allele a significantly increased relapse risk was observed for the carriers of the *UGT2B7^268His ^*allele (Figure 2a; log-rank *P *= 0.017). The incidences of relapses and other events constituting a disease progression were 33.1% and 15.2% in carriers and non-carriers of the *UGT2B7^268His ^*allele, respectively. Accordingly, carriers of at least one *UGT2B7^268His ^*allele had a shorter mean invasive disease-free survival time of 7.5 years (95% CI 6.9 to 8.0) as compared to 8.6 years (95% CI 7.9 to 9.3) in *UGT2B7^268Tyr/Tyr ^*carriers (Figure 2a, Table 3). The unadjusted hazard ratio (HR) for carriers of at least one *UGT2B7^268His ^*allele was 2.24 (95% CI 1.13 to 4.44; *P *= 0.021). Cox's regression models with adjustment for age at diagnosis, nodal status and tumor grade, stratified by tumor size due to non proportional hazard, resulted in an only slightly deviating adjusted HR of 2.64 (95% CI 1.22 to 5.71; *P *= 0.014).

**Table 3:** Invasive disease-free survival according to *UGT2B7^His268Tyr ^*genotype and treatment

	Hazard ratio (95% CI)	
Adjuvant chemotherapy	Crude	Adjusted
Epirubicin	2.24 (1.13 to 4.44)	2.64 (1.22 to 5.71)
	*P = 0.021*	*P = 0.014*
Epirubicin/tamoxifen	4.43 (1.34 to 14.6)	5.22 (1.67-26.04)
	*P = 0.015*	*P = 0.015*)
No epirubicin	0.80 (0.43 to 1.49)	0.78 (0.40 to 1.50)
	*P = 0.48*	*P = 0.45*
None	0.80 (0.47 to 1.34)	0.87 (0.49 to 1.55)
	*P = 0.39*	*P = 0.64*

CI, confidence interval.

Interestingly, in the subgroup of patients subsequently treated with tamoxifen the effect of the *UGT2B7^268His ^*allele to indicate a poor prognosis became even more pronounced (Figure 2b, Table 3) with an adjusted HR of 5.22 (95% CI 1.67 to 26.04; *P *= 0.015). However, in this model the Firth correction was applied as all patients with nodal status 0 were censored [28].

**Figure 3:**
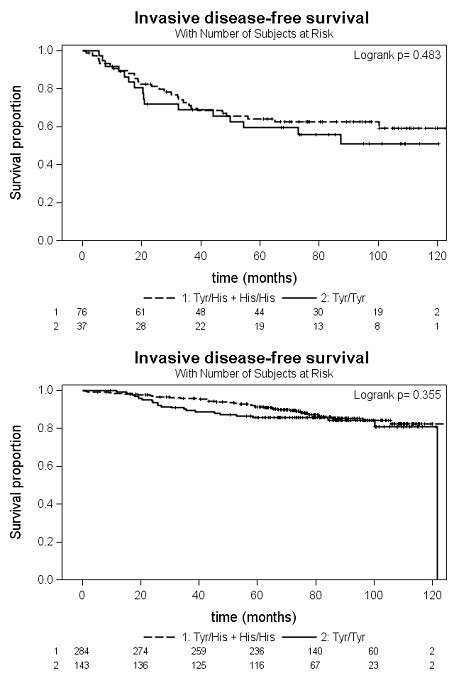
**Kaplan-Meier plots of invasive disease-free survival depending on the *UGT2B7^His268Tyr ^*genotype in control patients**. (a) Patients receiving a combination chemotherapy not containing epirubicin or (b) no adjuvant chemotherapy at all.

In the patient subgroup not receiving epirubicin, but tamoxifen in monotherapy (*n *= 305), there was no association of invasive disease-free survival with the *UGT2B7^His268Tyr ^*genotype (log-rank *P *= 0.20), as well as in any other patient group not receiving epirubicin (Figure 3, Table 3).

## Discussion

Genetic polymorphisms that might be relevant to drug metabolism and elimination have extensively been studied over the last years to explain between-subject variation in drug responses and to better target corresponding treatments [29-32]. Particularly in breast cancer, genetic polymorphisms of proteins involved in drug transport or metabolism have been shown to affect the efficacy of agents such as tamoxifen, taxanes or aromatase inhibitors [7,33,34]. A considerable variation in the response to epirubicin-based chemotherapies has been reported as well [35-37].

The formation of epirubicin glucuronide represent the main inactivating pathway for epirubicin and studies in human liver microsomes expressing a multitude of UGT isoenzymes, revealed that UGT2B7 uniquely converts epirubicin to its glucuronide [6]. To the best of our knowledge, this is the first study providing evidence that genetic *UGT2B7 *variation contributes to these clinical observations. In our analysis, epirubicin-treated women with the *UGT2B7^268Tyr/Tyr ^*genotype showed a significantly better invasive disease-free survival as compared to carriers of at least one *UGT2B7^268His ^*allele. As the use of epirubicin is quite common and the difference in the average invasive disease-free survival times between both variant carriers amounted to about one year, we think this finding might be of broad interest and high relevance in cancer therapy, although it still needs to be confirmed.

A lower glucuronidation capacity of the UGT2B7^268Tyr ^isoform may delay elimination and cause higher exposure to epirubicin and its active metabolites. This may eventually result in the observed longer invasive disease-free survival of carriers of the *UGT2B7^268Tyr/Tyr ^*genotype. This is in line with biochemical studies revealing a decreased activity of the UGT2B7^268Tyr ^isoforms assessing other UGT2B7 substrates [8-12]. However, those were contradicted by others [13-18] and by two *in vitro *studies showing no impact of the *UGT2B7^His268Tyr ^*genotype on the epirubicin glucuronide formation [6,19]. One of these studies utilized two lines of HK293 cells expressing the two isoforms generated via site-directed mutagenesis [6]. This methodology implies the disadvantage that the variant leading to an amino acid exchange is solely studied, whereas potentially linked variants remain unconsidered [6,16]. This seems to be important, since the *UGT2B7^His268Tyr ^*variant appears in frequent haplotypes with other variants, especially promoter variants (-1306 G > A, -1299C > T, -1112 C > T, -900 A > G, -327 G > A and -161 C > T) which seem to influence the expression of the two UGT2B7^His268Tyr ^isoforms [13,19-22]. The variant alleles of those promoter variants were associated with a two-fold decreased promoter activity and are perfectly linked with the *UGT2B7^268Tyr ^*allele [22]. Djebli *et al*. genotyped the -900 A > G promoter variant beside *UGT2B7^His268Tyr ^*and they reported that all individuals carrying the -900GG genotype carried also two variant *UGT2B7^268Tyr ^*alleles and those who were heterozygote for the promoter variant were as well heterozygote for *UGT2B7^His268Tyr ^*[21]. Using the tagging algorithm implemented in Haploview and the genotype frequencies for the HapMap CEU samples we observed that *UGT2B7^His268Tyr ^*and the linked promoter variants captured 83% of all genotyped markers (applying a r^2 ^cut-off of 0.8, minor allele frequency > 0.05, and using NM_001074 +/- 10 kb) [38,39]. The *UGT2B7^His268Tyr ^*variant is further linked to the rather rare promoter variant -138 G > A (less than 2% in Caucasians) which was associated with a seven-fold decreased promoter activity and it cannot be excluded if this variant contributes to our observations [22]. The presently available *in vitro *studies cannot finally clarify, if those promoter variants may modulate up- or downregulation of the enzyme, especially under the conditions of adjuvant or neoadjuvant chemotherapy. Therefore, it remains unclear if our observations can be mainly attributed to the *UGT2B7^His268Tyr ^*variant or to the expressional regulation caused by linked promoter variants. However, in human liver microsomes, genotyped for *UGT2B7^His268Tyr^*, no difference related to this polymorphism in epirubicin glucuronide formation was observed [19], but it is conceivable that there is an up- or downregulation of UGT2B7 caused by anti-cancer drug therapy which may differ inter-individually depending on genetic variants. Whether or not this does play a relevant role in epirubicin treatment cannot finally be clarified by our study but may be an interesting topic for further research. Sawyer *et al*. also observed differences in morphine glucuronidation studying the linked *UGT2B7 -161 C > T *variant in pain patients, but failed to confirm their finding in genotyped microsomes [18]. This may indicate differences between the *in vitro *and the *in vivo *impact of the variant or the associated *UGT2B7 *haplotypes.

A few studies have investigated the impact of the *UGT2B7^268Tyr ^*variant on the *in vivo *metabolism of other drugs. For mycophenolic acid (MPA), a lower glucuronidation activity and MPA-acyl-glucuronide formation rate through the *UGT2B7^268Tyr ^*variant was shown [21,40], which resulted in fewer gastrointestinal side-effects among MPA treated patients [41]. However, in contrast to MPA-acyl-glucuronide [41,42], the glucuronide of epirubicin are not known to contribute considerably to the side effects of the parental drug. Moreover, changes in drug tolerability may affect efficacy outcomes only if those result in changes in treatment adherence. Actually, we have neither data on epirubicin dose modifications and premature discontinuations nor on the incidence of adverse drug reactions. Additionally, an association of the linked *UGT2B7 -161 C > T *variant with pharmacokinetics of lamotrigine in epileptic patients has been recently reported [43]. By contrast, no effect was observed either on pharmacokinetics of efavirenz or zidovudine in human immunodeficiency virus infected patients [44,45], or on the treatment response to various opioids [46-48]. Given that our study was a retrospective analysis, we unfortunately were not able to determine plasma levels of drug compounds or metabolites. A possible influencing factor might be the younger age among *UGT2B7^268Tyr/Tyr ^*carriers in the epirubicin treated patients group, but although age is a relevant covariate for survival, the relatively small difference in mean age between the different *UGT2B7 *genotypes is very unlikely to explain a major part of the relatively strong UGT2B7 effects on survival after epirubicin treatment. In addition, we observed no difference in patient age comparing the *UGT2B7 *genotype groups within the other patient subgroups and no association between age at diagnosis and outcome in the multivariate analysis. Besides, a link to breast cancer risk has also been discussed for the *UGT2B7^His268Tyr ^*polymorphism, but no clear evidence has been reported and it is very unlikely that this is a contributing factor to our observations [49,50].

In addition, the association of the *UGT2B7^268Tyr ^*variant with invasive disease-free survival was even more pronounced in the subgroup of epirubicin-treated women who subsequently received tamoxifen (Figure 2b). Actually, *in vitro *data suggested reduced glucuronidation rates of the active metabolites 4-hydroxy tamoxifen and endoxifen through the UGT2B7^268Tyr ^isoform [9], but in contrast to epirubicin, sulfotransferases also play a quantitatively important role [51]. Accordingly, no difference in steady state plasma concentrations of tamoxifen metabolites was observed in 240 breast cancer patients receiving 20 mg tamoxifen daily [52]. In the present study, the sequential treatment with two UGT2B7 substrates resulted in a considerable effect of the *UGT2B7^His268Tyr ^*variant, whereas there was no difference in invasive disease-free survival among patients not treated with epirubicin, including the subgroup of women treated with tamoxifen alone. The more pronounced effect of the *UGT2B7^His268Tyr ^*polymorphism in patients treated with both epirubicin and tamoxifen remains currently unexplained and is limited by the rather small sample size of this patient subgroup.

## Conclusions

Breast cancer patients carrying the *UGT2B7^268Tyr/Tyr ^*genotype may benefit most from adjuvant epirubicin-based chemotherapy. Prospective studies with pharmacokinetic measurements and fixed treatment regimens are warranted.

## Abbreviations

CA15-3: cancer antigen 15-3; CEA: carcinoembryonic antigen; CI: confidence interval; HK293: human kidney 293 cell line; HR: hazard ratio; MPA: mycophenolic acid; PCR: polymerase chain reaction; PGt: pharmacogenetic; SD: standard deviation; TIGER: Austrian tumor of breast tissue: incidence, genetics, and environmental risk factors study; UDP: uridine diphosphate; UGT: UDP-glucuronosyltransferase.

## Competing interests

The authors declare that they have no competing interests.

## Authors' contributions

JCS, WR and EHB conceived the idea for the present analysis and designed the study. WR provided the study material. SP, JCS, AHW, UL, WR, PK and EHB collected the data. SP, JCS, AK, JB, AHW, UL, WR, PK and EHB analyzed and interpreted the data. SP, JCS and EHB prepared the manuscript. All authors revised the manuscript and gave their final approval.
